# Practical Points in Nursing, for Nurses in Private Practice

**Published:** 1896-10

**Authors:** 


Practical Points in Nursing, for Nurses in Private Practice. By Emily A. M. Stoney, Graduate of the training school for nurses, Lawrence, Mass. ; Superintendent of training school for nurses, Carney Hospital, South Boston, Mass. Octavo, pp. 456. Illustrated. Price, $1.75 net. Philadelphia: W. B. Saunders, 925 Walnut street. 1896.

The author has written one of the best books on nursing that we have seen. It does not aim to take the place of a text-book on nursing for use in training schools, but to the graduate just entering upon private nursing, and to the home nurse, the work, replete in suggestions as to how to improvise the many sick-room necessities and how to meet the various emergencies which arise, should be invaluable. Not all the emergency treatment suggested is wise, as, for instance, the method of dealing with foreign bodies in the ear, but on the whole the nurse may safely follow the author's instructions.

The appendix includes a dose list and an excellent glossary.

